# Lactic Acid Metabolism and Transporter Related Three Genes Predict the Prognosis of Patients with Clear Cell Renal Cell Carcinoma

**DOI:** 10.3390/genes13040620

**Published:** 2022-03-30

**Authors:** Tuanjie Guo, Jian Zhang, Tao Wang, Zhihao Yuan, Heting Tang, Dongliang Zhang, Siteng Chen, Xiang Wang

**Affiliations:** 1 Department of Urology, Shanghai General Hospital, Shanghai Jiao Tong University School of Medicine, Shanghai 200080, China; tuanjie-guo@sjtu.edu.cn (T.G.); jian.zhang2@shgh.cn (J.Z.); wtpaul@163.com (T.W.); zhihao_yuan@outlook.com (Z.Y.); cranetht@sjtu.edu.cn (H.T.); zhangdlmn@163.com (D.Z.); 2 Graduate School, Shanghai Jiao Tong University School of Medicine, Shanghai 200025, China

**Keywords:** clear cell renal cell carcinoma, lactic acid metabolism and transporter, three genes, prognosis, cell cycle

## Abstract

Lactic acid was previously considered a waste product of glycolysis, and has now become a key metabolite for cancer development, maintenance and metastasis. So far, numerous studies have confirmed that tumor lactic acid levels are associated with increased metastasis, tumor recurrence and poor prognosis. However, the prognostic value of lactic acid metabolism and transporter related genes in patients with clear cell renal cell carcinoma has not been explored. We selected lactic acid metabolism and transporter related twenty-one genes for LASSO cox regression analysis in the E-MTAB-1980 cohort, and finally screened three genes (*PNKD*, *SLC16A8*, *SLC5A8*) to construct a clinical prognostic model for patients with clear cell renal cell carcinoma. Based on the prognostic model we constructed, the over survival (hazard ratio = 4.117, 95% CI: 1.810–9.362, *p* < 0.0001) of patients in the high-risk group and the low-risk group in the training set E-MTAB-1980 cohort had significant differences, and similar results (hazard ratio = 1.909, 95% CI: 1.414–2.579 *p* < 0.0001) were also observed in the validation set TGCA cohort. Using the CIBERSORT algorithm to analyze the differences in immune cell infiltration in different risk groups, we found that dendritic cells, M1 macrophages, and CD4^+^ memory cells in the high-risk group were significantly lower than those in the low-risk group, while Treg cells were higher than in the low-risk group. Finally, through gene enrichment analysis, we found that the signal pathway that is strongly related to the prognostic model is the cell cycle.

## 1. Introduction

As early as 1920, Otto Warburg had discovered that cancer cells obtain a large proportion of energy through glycolysis even when oxygen is sufficient. Cancer cells can not only obtain energy through aerobic glycolysis, but also obtain energy through glutamine metabolism [1]. Cancer cells generate a large amount of lactic acid while obtaining energy through these two ways, which creates an acidic environment for the tumor microenvironment (TME). Studies have proven that the large accumulation of lactic acid in TME not only promotes tumor growth and metastasis, but also inhibits the anti-tumor immunity of immune cells [2]. The metabolic disorder of lactic acid is closely related to the abnormal expression of the enzymes that produce and transport lactic acid [3,4]. High expression of genes and proteins related to lactic acid metabolism and transporters often indicates high invasion and poor prognosis [5,6].

Clear cell renal cell carcinoma (ccRCC), as a highly aggressive malignant tumor, has a variety of metabolic disorders in TME. CcRCC is usually accompanied by the reprogramming of glucose, fatty acid metabolism and the tricarboxylic acid cycle. In addition, the metabolism of some amino acids such as tryptophan, arginine and glutamine is also reprogrammed in ccRCC [7]. Studies have also shown that kidney cancer cells can promote tumor progression by decomposing fat around the kidney to produce lactic acid [8]. However, the prognostic role of lactic acid metabolism and transporter related genes in patients with clear cell renal cell carcinoma remains unclear.

**Figure 1:**
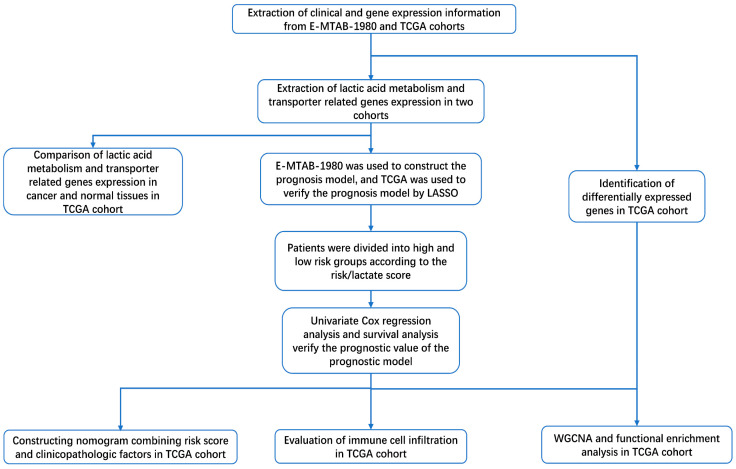
The workflow of this study.

In this study, we used lactic acid metabolism and transporter related genes to construct a prognostic model, which is closely related to the survival and prognosis of patients. We found that the infiltration of immune cells in two risk groups is different. Finally, we also found that the underlying mechanism of this model to predict prognosis is related to the cell cycle signaling pathway. The workflow of this study is shown in Figure 1.

## 2. Materials and Methods

### 2.1. Patient Cohorts and Data Sources

In this study, we included two cohorts, the training set E-MTAB-1980 (https://www.ebi.ac.uk/, accessed on 7 November 2021) cohort and the validation set, The Cancer Genome Atlas (TCGA, https://portal.gdc.cancer.gov/, accessed on 7 November 2021) cohort [9]. Only patients with complete mRNA expression and clinical information were included in the study, otherwise they would be excluded. In the E-MTAB-1980 cohort, a total of 101 ccRCC patients’ mRNA sequencing data and corresponding clinical information were extracted from the E-MTAB-1980 database, their gene expression data were standardized by the uploader. A total of 526 patients in the TCGA cohort were included in the study. Their mRNA sequencing data and clinical information were downloaded through the TCGA database, and all gene expression data were standardized before use. The statistical description of the clinical information of the patients in the two cohorts is detailed in Table 1. Simultaneously, 21 lactic acid metabolism and transporter related genes were obtained from GSEA (http://www.gsea-msigdb.org, accessed on 7 November 2021), and all genes can be found in the Supplementary Data S1.

### 2.2. Establishment and Verification of Prognostic Model

First of all, we extracted the mRNA expression of 21 lactic acid metabolism and transporter related genes and the clinical information of the patients in the E-MTAB-1980 data set. Subsequently, based on the glmnet package in the R language environment, the least absolute contraction and selection operator (LASSO)-cox regression analysis was carried out in order to screen out the genes most relevant to the prognosis. We set lambda to 1000 when calculating the coefficients to ensure the robustness of the model. The formula for risk score is as follows: risk score = $\sum_{i=1}^{n}\left(\alpha \;i*exp\;i\right)$. In this formula, *n* represents the number of prognostic genes, i represents the prognostic genes, *α* represents the coefficient of the i gene, and *exp* represents the mRNA expression value of the i gene. According to the median of risk score, patients were divided into a high-risk group and a low-risk group. Subsequently, we used the same method to validate this prognostic model in the TCGA cohort. 

### 2.3. Univariate Cox Regression Analysis and Survival Analysis

Cox regression analysis was implemented through the dplyr and survival packages. The survival analysis in this study was implemented through survival and survminer packages. Data visualizations were all exported from R software.

### 2.4. Constructing Nomogram Combining Risk Score and Clinicopathologic Factors

We constructed the predictive nomogram of ccRCC patients, and we combined risk score with clinicopathological factors by nomogramEx and rms package. Receiver operating characteristic (ROC) curve and decision-making curve with area under curve (AUC) values were further calculated to evaluate the prediction of the nomogram.

### 2.5. Immune Cell Infiltration in Tumor Microenvironment

The CIBERSORT algorithm was used to evaluate the difference in immune cell infiltration between the high-risk group and the low-risk group of ccRCC patients, and to explore the immunological mechanism of the prognosis model. According to the instructions [10], the LM22 file was used as the signature of immune cells, and the permutations were set as 1000. All immune cells proportion analysis processes were implemented in the R environment.

### 2.6. Identification of Differentially Expressed Genes

We used the edger package to normalize the gene count value, and then used the limma package to select genes with |log_2_FC| ≥ 1 and FDR < 0.05 in the TCGA database.

### 2.7. Functional Enrichment Analysis

In order to explore the potential mechanism by which our prognostic model can predict the prognosis of ccRCC patients, we performed a weighted gene co-expression network analysis (WGCNA) using the previously identified differentially expressed genes in cancer and adjacent normal tissues. Whereafter, we selected the gene module most relevant to the risk score, extracted the genes from the gene module and performed the Kyoto Encyclopedia of Genes and Genomes (KEGG) approach and genetic ontology (GO) analysis in Metascape [11].

### 2.8. Statistical Analysis

In this study, the Mann-Whitney U test was performed to compare continuous variable. Kaplan–Meier curve analysis was carried out by using a log-rank test to compare OS. Cox regression analysis was performed to explore whether risk score could act as a prognostic factor for ccRCC. All statistical analyses were performed in R (4.0.0) and GraphPad Prism 8.

## 3. Results

### 3.1. Identification of Lactic Acid Metabolism and Transporter Related Three Genes as Prognostic Model of ccRCC Patient

**Figure 2:**
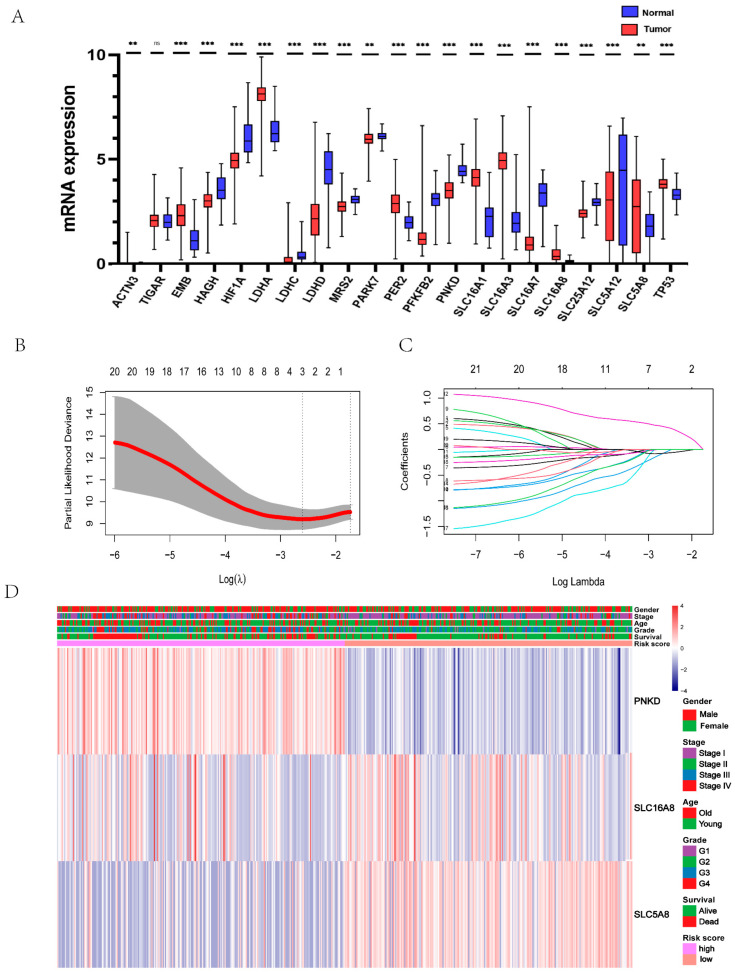
Establishment of prognostic model. (**A**) Differential mRNA expressions of lactate metabolism and transport related genes between clear cell renal cell carcinoma and normal renal tissue. (**B**,**C**) The tenfold cross-validated error and coefficients at varying levels of penalization plotted against the log (lambda) sequence for the least absolute shrinkage and selection operator analysis. (**D**) Heatmap illustrated the expression of the selected genes and the distribution of clinicopathologic factors in the TCGA cohort. ccRCC, clear cell renal cell carcinoma; TCGA, the cancer genome atlas. **: *p* < 0.01; ***: *p* < 0.001; ns: no significance.

Given that lactic acid metabolism is disordered in the TME, we first compared whether there were significant differences in the expression of lactic acid metabolism and transporter related genes in clear cell renal cell carcinoma tissues and normal adjacent tissues. Among all 21 lactic acid metabolism and transporter related genes, 20 genes have significant expression differences. Compared with normal tissues, nine genes are up-regulated and 11 genes are down-regulated in cancer tissues (Figure 2A), which is consistent with lactic acid metabolism disorder [12]. In order to make the prognostic model more robust, we used a small sample of E-MTAB-1980 cohort as a training set to construct a prognostic model, and a total of three genes (*PNKD*, *SLC16A8*, *SLC5A8*) were screened to construct the model (Figure 2B,C); their respective weights can be found in Supplementary Table S1. Finally, we integrated the selected genes and the clinical information of the patients into the heatmap for analysis, and the correlation between the risk score and gene expression and clinicopathological information can be intuitively observed (Figure 2D).

### 3.2. The Constructed Prognostic Model Can Be Used as a Prognostic Factor

**Figure 3:**
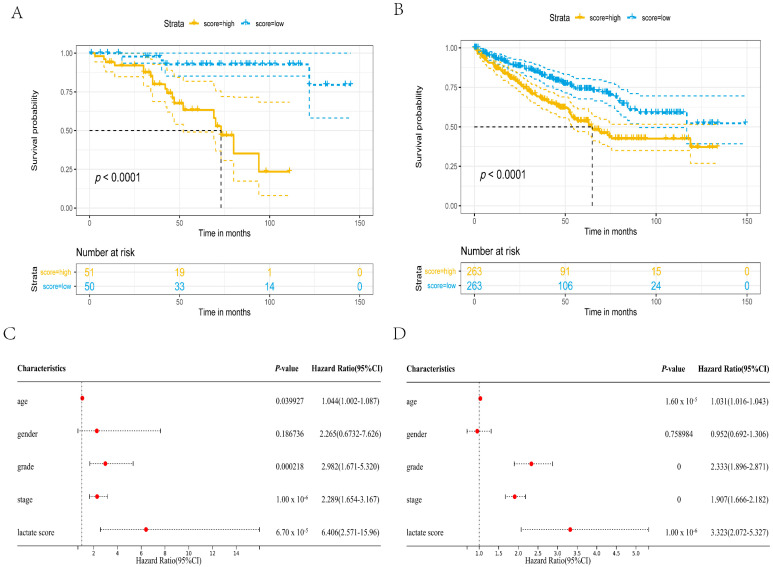
The three-gene prognostic model related to lactate metabolism and transporter can be used as a prognostic factor. (**A**) Kaplan–Meier survival analysis of overall survival stratified by risk score for ccRCC patients in the E-MTAB-1980 cohort. (**B**) Kaplan–Meier survival analysis to validate the prognostic model in the TCGA cohort. (**C**) Univariate Cox regression analysis of the E-MTAB-1980 cohort. (**D**) Univariate Cox regression analysis of the TCGA cohort.

Subsequently, we used the aforementioned formula to calculate the risk score of each patient in the E-MTAB-1980 cohort, and divided them into a high-risk group and a low-risk group (high score means high risk) based on the median score for survival analysis. There is a significant difference in OS (hazard ratio = 4.117, 95% CI: 1.810–9.362, *p* < 0.0001) between the two groups of patients, and patients in the high-risk group have a lower survival rate (Figure 3A). Homoplastically, in the TCGA cohort, it can be observed that the OS (hazard ratio = 1.909, 95% CI: 1.414–2.579, *p* < 0.0001) of ccRCC patients has a similar result (Figure 3B). Considering that the risk score can significantly distinguish the survival rate and clinicopathological information of ccRCC patients, we want to know whether the risk score can be used as a prognostic factor for ccRCC patients. Therefore, we performed univariate Cox regression analysis in two independent cohorts, and the hazard ratio of the risk score in the E-MTAB-1980 cohort was 6.406, *p* < 0.0001 (Figure 3C). Simultaneously, we also obtained an analogous conclusion in the TCGA cohort (Figure 3D). In summary, we can conclude that the risk score of lactic acid metabolism and transporter can be used as a prognostic factor for ccRCC patients. 

### 3.3. Risk Score Is Strongly Correlated with the Patient’s Clinicopathological Information

**Figure 4:**
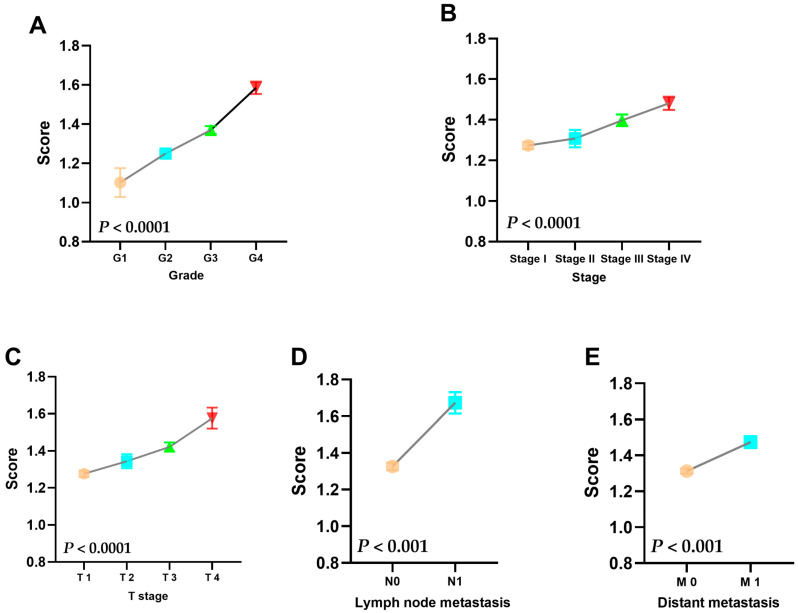
Relationship between risk score and different clinicopathological information groups based on TCGA cohort. (**A**) The relationship between risk score and Grade. (**B**) The relationship between risk score and Stage. (**C**) The relationship between risk score and T stage. (**D**) The relationship between risk score and lymph node metastasis. (**E**) The relationship between risk score and distant metastasis.

We want to know whether there is a difference in risk scores in different subgroups of clinicopathology information. In the TCGA cohort, we found that the risk score gradually increased as the Grade increased (Figure 4A). Similarly, the patient’s risk score will also gradually increase with the increase of Stage (Figure 4B). The T stage of renal cell carcinoma mainly represents the size of the tumor, and the risk score also increases as the tumor increases (Figure 4C). In addition, the most important thing is that when the risk score increases, the risk of tumor metastasis also gradually increases (Figure 4D,E). Taken together, the higher the risk score, the more likely the tumor is to develop into a serious clinical pathological state. It can be explained that our prognostic model is strongly related to clinicopathological information.

### 3.4. Nomogram Based on Prognostic Model and Clinicopathological Information can Predict the Prognosis of ccRCC Patients

**Figure 5:**
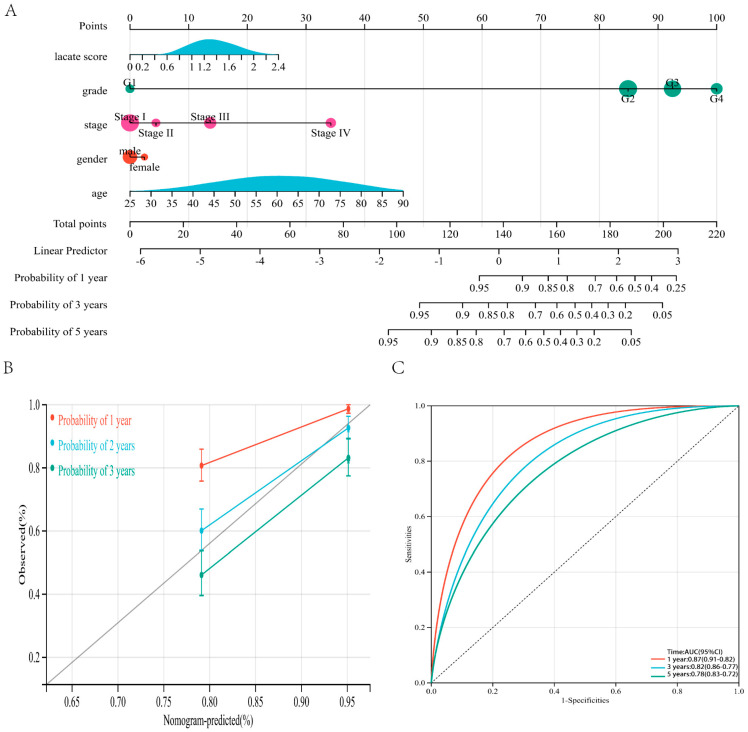
Nomogram prediction model can accurately predict the prognosis of patients. (**A**,**B**) Nomogram based on prognostic model and clinicopathological information. (**C**) ROC curve of the TCGA cohort.

In order to combine the prognostic model with the clinicopathological information of patients to predict the survival probability of patients, we constructed a nomogram prediction model (Figure 5A,B). The survival prediction accuracy of the nomogram for patients with 1-, 3- and 5-years was 0.87, 0.82, and 0.78, respectively (Figure 5C). Therefore, the prognostic model we established can be used as a significant factor for predicting the survival of ccRCC patients.

### 3.5. Prognostic Model Is Associated with Immune Cell Infiltration

**Figure 6:**
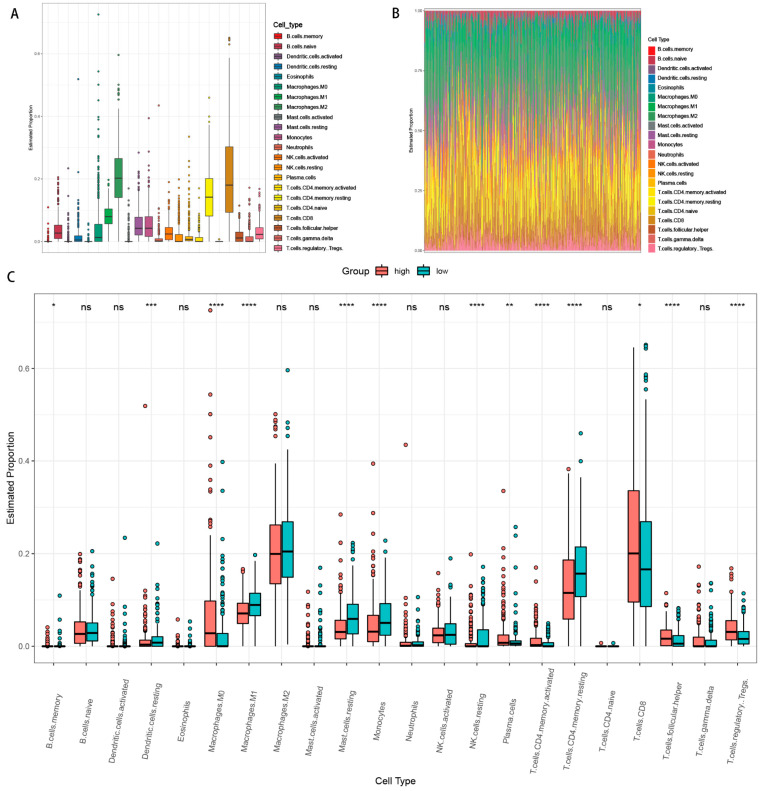
Distribution of immune cells in tumor microenvironment in the TCGA cohort. (**A**) Each type of immune cell accounts for information in the tumor microenvironment. (**B**) The proportion of immune cells in each patient in the TCGA cohort. (**C**) The difference in the proportion of immune cells between the high-risk group and the low-wind group. *: *p* < 0.05; **: *p* < 0.01; ***: *p* < 0.001; ****: *p* < 0.0001; ns: no significance.

Lactic acid metabolism can affect the distribution and phenotype of immune cells [13]. In order to explore whether there are significant statistical differences in the proportion of immune cells in patients with different risk groups, we used the CIBERSORT algorithm for analysis. In the TME of ccRCC patients, we found that the top three immune cells with the largest proportions are M2 type macrophages, CD4^+^ T memory cells, and CD8^+^ T cells (Figure 6A). So as to show the proportion of immune cells in each patient more intuitively, we visualized the proportion of immune cells in each patient (Figure 6B). To further study the relationship between our risk model and immune cell infiltration, we analyzed the differences in the immune infiltration of ccRCC patients between high-risk and low-risk groups (Figure 6C). We found that the tumors of patients in the high-risk group had more Treg cells and macrophages, and fewer dendritic cells, M1 macrophages, and CD8^+^ T cells than those in the low-risk group. This may be related to the worse prognosis of ccRCC patients with higher CD8^+^ T cell infiltration [14]. In addition, it may also be related to the ability of lactic acid to supply Treg cell metabolism and proliferation [15]. In short, for the first time, we established a prognostic model based on lactic acid metabolism and transporter related genes to analyze the infiltration of immune cells in the TME.

### 3.6. WGCNA Reveals That High Risk Score Is Related to Cell Cycle

**Figure 7:**
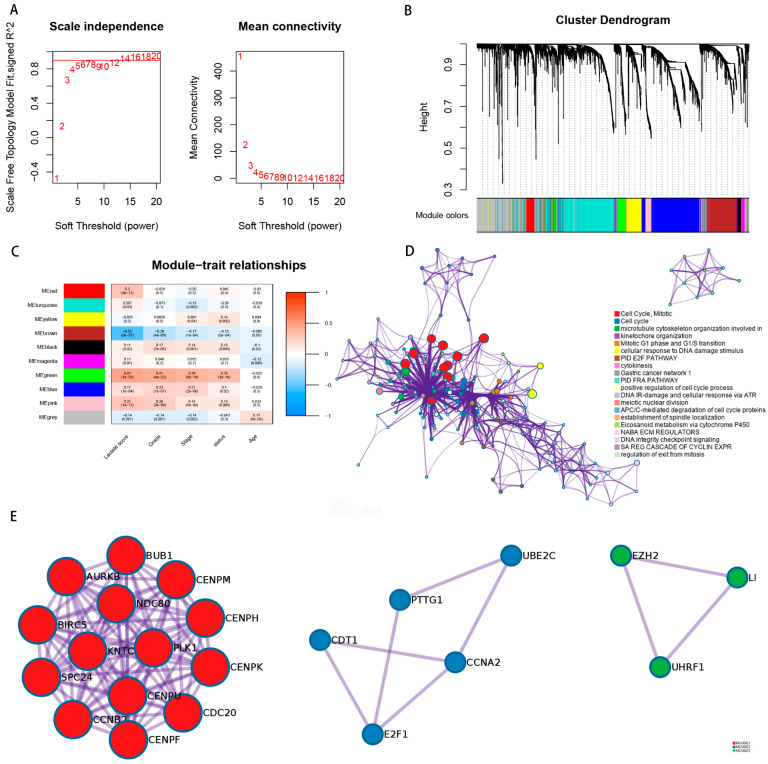
Gene weight co-expression network analysis and gene function enrichment analysis. (**A**) Soft power estimation in ccRCC for WGCNA. (**B**) Gene dendrogram with different colors showing the modules identified by WGCNA. (**C**) The relationship between gene modules and clinical characteristic. (**D**) Potentially enriched pathways of the co-expressed genes in green module. (**E**) The protein–protein interaction network of the top three signaling pathways.

Finally, in order to explore the potential mechanism of our model for predicting prognosis, we performed a weighted gene co-expression network analysis in the TCGA cohort based on the previously identified differentially expressed genes. We analyzed 2936 differentially expressed genes in the TCGA cohort through WGCNA. According to the suggestion of pickSoftThreshold, the soft threshold power of the β value was set to 14 (Figure 7A). Subsequently, all genes related to ccRCC were hierarchically clustered into 10 gene modules (Figure 7B). The correlation analysis shows that the green model (MEgreen) has the highest correlation with the risk score (Figure 7C). Then we performed a functional enrichment analysis of the genes in the green module, and we found that the most relevant signal pathway for risk score is the cell cycle (Figure 7D). Ultimately, we performed protein–protein interaction analysis (Figure 7E) on the genes in the first three signal pathways of Figure 7D.

## 4. Discussion

Clear cell renal cell carcinoma is the most common malignant tumor in kidney tumors. The prognosis of patients with tumors that can be completely removed by surgery is good. At present, the most commonly used factors that can be used as a prognostic reference for patients are mainly TNM stage and Fuhrman grade system, but there are no other reference factors that can guide the prognosis [16]. Therefore, it is urgent to develop a tool that can predict the prognosis of ccRCC patients. 

We build a prognostic model based on the small sample E-MTAB-1980 cohort as the training set, and then validate the model on the large sample TCGA cohort, which makes our model more convincing. The constructed model can clearly separate the survival curves of patients in the high-risk and low-risk groups in the two cohorts, and the differences are statistically significant, which fully demonstrates that the model is powerful and convincing. In addition, the patient’s Stage, Grade, T stage, lymph node metastasis and distant metastasis are all associated with the risk score, and the score will increase as the disease progresses. Nevertheless, the model we built can also accurately predict the survival time of patients, which fully affirms the important value of our model and the possibility of clinical use. 

Excessive lactic acid production by cancer cells is related to the abnormal expression of lactic acid metabolism enzymes [13]. The effect of lactic acid on immune cells has gradually become apparent. Above all, it can induce M2-like polarization of tumor-associated macrophages. The presence of lactic acid alone can induce the expression of VEGF and ARG1 in macrophages, thereby promoting tumor growth and metastasis [17]. This is consistent with our previous conclusion that M2 macrophages account for a large proportion of the tumor microenvironment in ccRCC patients. Lactic acid can inhibit the differentiation of monocytes into dendritic cells. Higher lactic acid levels in the tumor microenvironment may hinder the formation and accumulation of dendritic cells [13,18,19]. This is consistent with our analysis that the high-risk group had a lower percentage of dendritic cells. In addition, the lactic acid in the tumor microenvironment can make CD8^+^ T cells highly express PD-1 and reduce the secretion ability of cytokines [20]. More importantly, Treg cells can use lactic acid as a source of nutrients to promote their own proliferation and differentiation [15]. Interestingly, we also observed a higher proportion of Treg cells in patients in the high-risk group. It can be explained that the poor prognosis of patients in the high-risk group is related to the suppression of anti-tumor immunity function.

The accumulation of lactic acid in TME is not only due to the high expression of lactic acid metabolizing enzymes, but the lactic acid transporter also plays an indispensable role in it. When the lactic acid transporter is highly expressed, it can significantly increase the invasion and metastasis ability of tumor cells, and inhibiting the lactic acid transporter can reduce the proliferation and metastasis ability of cancer cells [5,21]. The latest research has found that the use of small molecule targeted inhibitors of lactic acid transporters can reverse the immunosuppressive microenvironment of solid tumors and improve the safety and anti-tumor efficacy of tumor immunotherapy [22,23,24]. Blocking lactic acid metabolism and transporters is a very promising treatment for cancer.

Gene enrichment analysis found that the constructed prognostic model is strongly related to the cell cycle signaling pathway, and the Grade and Stage also increase with the increase of the risk score. The evaluation criterion of the Fuhrman grade system mainly depends on the cell division. The stage is mainly judged based on the size of the tumor, which happens to depend on the cell cycle. This is reflected in the accumulation of lactic acid in the TME to promote tumor proliferation. Based on the cell cycle signaling pathway, we also showed the protein–protein interaction network, suggesting that the risk score is related to the interaction of these proteins.

The three genes screened by our prognostic model have almost no researchers reporting their role in tumorigenesis and development. Among them, the *PNKD* gene has only been reported in Tourette Disorder or Tic disorder [25]. The *SLC16A8* gene has not been found to be associated with the disease. The biological behavior of *SLC5A8* in cervical cancer has been reported, which can alleviate the progression of cervical cancer by regulating the Wnt signaling pathway [26]. These three genes have greater research space in the future, and this will also be the direction and focus of our research.

We innovatively used lactic acid metabolism and transporter related genes to construct a prognostic model for ccRCC patients, and carefully analyzed the role of lactic acid-related genes in the prognosis of cancer patients. Although our model is excellent, it is inevitable that there are certain shortcomings. For example, the cohorts we use are all public databases, there will inevitably be some unknown factors that affect the results. In addition, there are still some unavoidable deviations in our data analysis process. Even though the model we established still has very important clinical significance, the real application of this prognostic model in clinical practice still requires large-scale prospective multicenter clinical studies.

## 5. Conclusions

At present, the prognosis of renal cell carcinoma is only dependent on the TNM stage and Fuhrman nuclear grading system. Our model can evaluate the prognosis of patients according to the expression of these three genes selected in the tumor tissue of patients after surgery. In conclusion, we used lactic acid metabolism and transporter related genes to construct a clinical prognosis model for ccRCC patients. The model can predict the prognosis of patients well, and the mechanism for predicting the prognosis of this model is related to the cell cycle. 

## Figures and Tables

**Table 1:** Basic clinical characteristics of patients in the TCGA cohort and E-MTAB-1980 cohort.

	TCGA Cohort (526)	E-MTAB-1980 Cohort (101)
Age (years)		
≥65	194 (36.9%)	44 (43.6%)
<65	332 (63.1%)	57 (56.4%)
Sex		
Male	343 (65.2%)	77 (23.8%)
Female	183 (34.8%)	24 (23.8%
Grade		
G1	13 (2.5%)	13 (12.9%)
G2	224 (42.6%)	59 (58.4%)
G3	204 (38.8%)	22 (21.8%)
G4	74 (14.1%)	5 (5.0%)
Unknown	8 (1.5%)	2 (2.0%)
Stage		
I	261 (49.6%)	67 (66.3%)
II	57 (10.8%)	11 (10.9%)
III	125 (23.8%)	14 (13.9%)
IV	83 (15.8%)	13 (12.9%)
T stage		
T1	267 (50.8%)	68 (67.3%)
T2	69 (13.1%)	11 (10.9%)
T3	179 (34.0%)	21 (20.85)
T4	11 (2.1%)	1 (1.0%)
N stage		
N2	\	4 (4%)
N1	16 (3.1%)	3 (3.0%)
N0	238 (45.2%)	94 (93.1%)
Unknown	272 (51.7%)	/
M stage		
M1	78 (14.8%)	12 (11.9%)
M0	418 (79.5%)	89 (88.1%)
Unknown	30 (5.7%)	/
Survival		
Dead	171 (32.5%)	23 (22.8%)
Living	355 (67.5%)	78 (77.25)

## Data Availability

In this study, we included two public datasets, which are the E-MTAB-1980 (https://www.ebi.ac.uk/, accessed on 7 November 2021) and The Cancer Genome Atlas (TCGA, https://portal.gdc.cancer.gov/, accessed on 7 November 2021). The datasets used and analyzed in this study are also available from the corresponding author upon reasonable request.

## Supplementary Materials

The following are available online at https://www.mdpi.com/article/10.3390/genes13040620/s1. Data S1: Lactate metabolism and transporter related genes. Table S1: Selected genes and associated weights in the prognosis model.

## References

[1] Warburg O. (1956). On the Origin of Cancer Cells. Science.

[2] Ippolito L., Morandi A., Giannoni E., Chiarugi P. (2019). Lactate: A Metabolic Driver in the Tumour Landscape. Trends Biochem. Sci..

[3] Pereira-Nunes A., Afonso J., Granja S., Baltazar F. (2020). Lactate and lactate transporters as key players in the maintenance of the warburg effect. Adv. Exp. Med. Biol..

[4] Halestrap A.P. (2013). The SLC16 gene family—Structure, role and regulation in health and disease. Mol. Asp. Med..

[5] Zhang G., Zhang Y., Dong D., Wang F., Ma X., Guan F., Sun L. (2018). MCT1 regulates aggressive and metabolic phenotypes in bladder cancer. J. Cancer.

[6] San-Millán I., Brooks G.A. (2016). Reexamining cancer metabolism: Lactate production for carcinogenesis could be the purpose and explanation of the Warburg Effect. Carcinogenesis.

[7] Wettersten H.I., Aboud O.A., Lara P.N., Weiss R.H. (2017). Metabolic reprogramming in clear cell renal cell carcinoma. Nat. Rev. Nephrol..

[8] Wei G., Sun H., Dong K., Hu L., Wang Q., Zhuang Q., Zhu Y., Zhang X., Shao Y., Tang H. (2021). The thermogenic activity of adjacent adipocytes fuels the progression of ccRCC and compromises anti-tumor therapeutic efficacy. Cell Metab..

[9] Sato Y., Yoshizato T., Shiraishi Y., Maekawa S., Okuno Y., Kamura T., Shimamura T., Sato-Otsubo A., Nagae G., Suzuki H. (2013). Integrated molecular analysis of clear-cell renal cell carcinoma. Nat. Genet..

[10] Newman A.M., Liu C.L., Green M.R., Gentles A.J., Feng W., Xu Y., Hoang C.D., Diehn M., Alizadeh A.A. (2015). Robust enumeration of cell subsets from tissue expression profiles. Nat. Methods.

[11] Zhou Y., Zhou B., Pache L., Chang M., Khodabakhshi A.H., Tanaseichuk O., Benner C., Chanda S.K. (2019). Metascape provides a biologist-oriented resource for the analysis of systems-level datasets. Nat. Commun..

[12] Hirschhaeuser F., Sattler U.G.A., Mueller-Klieser W. (2011). Lactate: A Metabolic Key Player in Cancer. Cancer Res..

[13] Certo M., Tsai C.-H., Pucino V., Ho P.-C., Mauro C. (2020). Lactate modulation of immune responses in inflammatory versus tumour microenvironments. Nat. Rev. Immunol..

[14] Fridman W.H., Zitvogel L., Sautes-Fridman C., Kroemer G. (2017). The immune contexture in cancer prognosis and treatment. Nat. Rev. Clin. Oncol..

[15] Watson M.J., Vignali P.D.A., Mullett S.J., Overacre-Delgoffe A.E., Peralta R.M., Grebinoski S., Menk A.V., Rittenhouse N.L., DePeaux K., Whetstone R.D. (2021). Metabolic support of tumour-infiltrating regulatory T cells by lactic acid. Nature.

[16] Zisman A., Pantuck A.J., Dorey F., Said J.W., Shvarts O., Quintana D., Gitlitz B.J., Dekernion J.B., Figlin R.A., Belldegrun A.S. (2001). Improved Prognostication of Renal Cell Carcinoma Using an Integrated Staging System. J. Clin. Oncol..

[17] Colegio O.R., Chu N.-Q., Szabo A.L., Chu T., Rhebergen A.M., Jairam V., Cyrus N., Brokowski C.E., Eisenbarth S.C., Phillips G.M. (2014). Functional polarization of tumour-associated macrophages by tumour-derived lactic acid. Nature.

[18] Gottfried E., Kunz-Schughart L., Ebner S., Mueller-Klieser W., Hoves S., Andreesen R., Mackensen A., Kreutz M. (2006). Tumor-derived lactic acid modulates dendritic cell activation and antigen expression. Blood.

[19] Puig-Kröger A., Pello O.M., Muñiz-Pello O., Selgas R., Criado G., Bajo M.A., Sánchez-Tomero J.A., Alvarez V., del Peso G., Sánchez-Mateos P. (2003). Peritoneal dialysis solutions inhibit the differentiation and maturation of human monocyte-derived dendritic cells: Effect of lactate and glucose-degradation products. J. Leukoc. Biol..

[20] Balgi A.D., Diering G.H., Donohue E., Lam K., Fonseca B.D., Zimmerman C., Numata M., Roberge M. (2011). Regulation of mTORC1 Signaling by pH. PLoS ONE.

[21] Guo C., Huang T., Wang Q.-H., Li H., Khanal A., Kang E.-H., Zhang W., Niu H.-T., Dong Z., Cao Y.-W. (2019). Monocarboxylate transporter 1 and monocarboxylate transporter 4 in cancer-endothelial co-culturing microenvironments promote proliferation, migration, and invasion of renal cancer cells. Cancer Cell Int..

[22] Huang T., Feng Q., Wang Z., Li W., Sun Z., Wilhelm J., Huang G., Vo T., Sumer B.D., Gao J. (2020). Tumor-Targeted Inhibition of Monocarboxylate Transporter 1 Improves T-Cell Immunotherapy of Solid Tumors. Adv. Healthc. Mater..

[23] Doherty J., Cleveland J.L. (2013). Targeting lactate metabolism for cancer therapeutics. J. Clin. Investig..

[24] Wang J.X., Choi S.Y., Niu X., Kang N., Xue H., Killam J., Wang Y. (2020). Lactic Acid and an Acidic Tumor Microenvironment suppress Anticancer Immunity. Int. J. Mol. Sci..

[25] Sun N., Nasello C., Deng L., Wang N., Zhang Y., Xu Z., Song Z., Kwan K., King R.A., Pang Z.P. (2017). The PNKD gene is associated with Tourette Disorder or Tic disorder in a multiplex family. Mol. Psychiatry.

[26] Zhang X.-M., Meng Q.-H., Kong F.-F., Wang K., Du L.-J. (2020). SLC5A8 regulates the biological behaviors of cervical cancer cells through mediating the Wnt signaling pathway. Eur. Rev. Med. Pharmacol. Sci..

