# Supplementary material for: Lactic Acid Metabolism and Transporter Related Three Genes Predict the Prognosis of Patients with Clear Cell Renal Cell Carcinoma
Source: Genes (Basel). 2022 Mar 30;13(4):620. doi: 10.3390/genes13040620 (PMC9032142; doi:10.3390/genes13040620)
Supplement: Supplementary file 1 [file genes-13-00620-s001.zip › genes-1640175-supplementary.pdf]

Supplementary Data S1: Lactate metabolism and transporter related genes.

PARK7
LDHD
PNKD
HAGH
HIF1A
LDHA
LDHC
PFKFB2
TIGAR
MRS2
TP53
SLC25A12
PER2
ACTN3
EMB
SLC5A12
SLC5A8
SLC16A8
SLC16A1
SLC16A3
SLC16A7

Supplementary Table S1: Selected genes and associated weights in the prognosis model.

Genes	Weights
PNKD	0.43603432
SLC16A8	-0.05299190
SLC5A8	-0.07168776
